# From Challenge to Cure: A Look at Feline Infectious Peritonitis and Emerging Treatment Strategies and Breakthroughs

**DOI:** 10.3390/vetsci12070650

**Published:** 2025-07-08

**Authors:** Sheema Mir, Mykah Peters, Gloria Penny, Alexis Agsaoa, Mohammad Mir

**Affiliations:** College of Veterinary Medicine, Western University of Health Sciences, Pomona, CA 91766, USAmmir@westernu.edu (M.M.)

**Keywords:** feline infectious peritonitis, feline coronavirus, mutated coronavirus, antiviral therapies, vaccination

## Abstract

Background: Feline infectious peritonitis (FIP) is a complex and devastating viral disease in cats caused by feline coronavirus (FCoV). While FCoV is commonly encountered and typically innocuous, the emergence of a mutated variant can lead to the development of FIP, a severe and often fatal condition. Method and Results: This review article provides a comprehensive overview of the etiological factors, epidemiology, clinical manifestations, and challenges associated with FIP. Additionally, it underscores the critical need for further research to enhance diagnostic capabilities and develop effective therapeutic interventions. Conclusion: By shedding light on the intricate dynamics of FIP, this review paper aims to contribute to a deeper understanding of the disease via fostering therapeutic advancements that can improve outcomes for afflicted felines.

## Simple Summary

Feline infectious peritonitis (FIP) is a deadly disease that affects cats and is caused by a mutation of feline coronavirus, a virus that many cats carry without ever falling seriously ill. In rare cases, the virus changes inside the cat’s body and leads to FIP, which is often fatal. This article looks at how FIP develops, how it spreads between cats, and why it is so hard to diagnose. It also covers the current tools and treatments available to help cats with FIP, though they are still limited. The review emphasizes how much more we need to learn about this disease and highlights the importance of research in finding better ways to detect, treat, and hopefully prevent FIP in the future.

## 1. Introduction

Feline infectious peritonitis (FIP) is a viral disease in cats caused by feline coronavirus (FCoV) [1]. FCoV is an enveloped, positive sense, single-stranded, non-segmented RNA virus [2]. The intricate interplay between feline coronavirus (FCoV) and its host manifests in the form of FIP, an ailment that exacts a heavy toll on the health of domestic cats and certain wild feline species [3]. FIP is a complex and often fatal disease that primarily affects young domestic cats and some wild feline species. While FCoV is typically widespread and innocuous, FCoV can take a menacing turn when subjected to genetic mutations, resulting in the development of FIP—a disease known for its complexity, high fatality rate, and elusive nature [4].

FIP was first recognized as a distinct disease in 1963 by Dr. Jean Holzworth and her team at Angell Memorial Animal Hospital in Boston, USA [1]. Unfortunately, specific global prevalence data for FIP by region is limited, as comprehensive, up-to-date studies on FIP prevalence across all global regions are rare. This review paper delves into the dynamics of FIP, exploring the underlying mechanisms that transform a seemingly benign coronavirus into a formidable pathogen. With a focus on the etiology, clinical manifestations, diagnostics, and therapeutics of FIP, we aim to provide a comprehensive understanding of the disease and its impact on feline health. Despite the widespread nature of FCoV, the development of FIP remains an enigma, often resulting in a dire prognosis for affected felines. In the following sections, we will discuss the key features of FIP, from its transmission patterns and risk factors to the challenges associated with diagnosis and treatment. As we navigate through the complexities of this viral disease, it becomes evident that a deeper comprehension is essential for the development of effective preventive strategies and therapeutic interventions. The urgency to unravel the mysteries surrounding FIP is underscored by its severe consequences, urging researchers and veterinarians alike to collaborate in the pursuit of mitigating the impact of this elusive and often fatal feline disease.

## 2. Pathogenesis of FIP

FIP is caused by a mutation of FCoV [5,6]. Most cats are exposed to the non-mutated form of the virus at some point in their lives, and a small percentage of these cats will develop FIP. The reasons behind the mutation and why some cats develop the disease while others do not are still not fully understood [2,7]. FCoV is transmitted via the fecal–oral route [8]. The virus enters and replicates in epithelial cells of small intestinal villi. Distinguished by serological variations, feline coronavirus (FCoV) strains are broadly classified into a prevalent type 1 form, detected in 80–90% of infected cats, and a less common type 2 form [9]. These two types are differentiated by their distinct cell entry receptors and unique in vitro growth characteristics, which are linked to variations in their spike genes [10,11]. Type 2 FCoV uses feline aminopeptidase-N receptor (fPAN) to invade small intestinal villi and monocytes. Type 1 FCoV processes of invasion are unknown [12,13]. The replication of FCoV in monocytes is a key event that leads to the development of FIP [14,15,16]. Usually when monocytes become infected, they will present viral antigens in association with a feline leucocyte antigen (feline version of histocompatibility complex) on its surface to allow antigen or cell mediate lysis. Monocytes infected with FIP lack surface expression and ultimately escape cell lysis [17,18,19,20]. Recent in vitro experiments involving chimeric viruses demonstrated that FCoV serotype 2 (strain 79-1146) and a chimera serotype 1 virus (strain Black), expressing a serotype 2 spike protein, engage the feline aminopeptidase N (fAPN) cellular receptor. Conversely, serotype 1 FCoVs likely use an alternative primary cellular receptor [21]. Furthermore, a feline counterpart to human dendritic cell-specific intercellular adhesion molecule 3-grabbing non-integrin (DC-SIGN), known as fDC-SIGN, acts as a coreceptor in both serotype 1 and 2 infections in vitro [22]. Both virulent and avirulent FCoV strains are found within types 1 and 2 in vivo [12].

According to the “internal mutation hypothesis”, also known as “in vivo mutation transition hypothesis”, “spontaneous virus mutation occurs in vivo, resulting in viruses capable of systemic spread and leading to the pathogenesis of Feline Infectious Peritonitis (FIP)” [23,24]. This widely accepted hypothesis, supported by numerous publications suggests that the specific mutation responsible for pathogenesis remains unidentified [13,23,25]. Studies propose the involvement of sequence differences in the spike protein [26,27], membrane protein [28], or NSP 3c [13]. In conjunction with in vitro findings describing the affinity of FIPV strains for macrophages compared to feline enteric coronavirus (FECV) strains [29], the hypothesis has been extended to suggest that the enteric coronavirus (FECV) undergoes a mutational shift in the gastrointestinal system. This shift allows the infection of macrophages, systemic dissemination, and the onset of fatal disease [23,24].

### 2.1. Clinical Signs of FIP

The replication of virulent FCoV strains within macrophages results in two forms of disease, which reflect the immune response mounted by the host [30]. These two forms are the wet (effusive) form and the dry (non-effusive) form. Clinical signs and physical examination findings in cats with FIP can indicate the specific form of the disease and the location of the lesions [30].

Non-specific signs of FIP include loss of appetite, lethargy, lymphadenopathy, weight loss, and a fever that usually does not respond to antibiotics [31]. The terminal stage of FIP includes findings like non-specific leukocytosis with a lymphopenia and a normocytic normochromic anemia [32].

### 2.2. Dry (Non-Effusive) Form

No fluid buildup but granuloma formation results in non-effusive (dry) FIP [5]. Signs are associated with the organs or tissues involved [33]. Organs that are especially involved include the mesenteric lymph nodes, kidneys, liver, lungs, brain, and eye [34]. Patients with FIP often exhibit effusions with granulomatous lesions at postmortem examinations [34,35].

Clinical signs seen in both forms of FIP include lethargy, anorexia, weight loss, pyrexia, and even jaundice [2]. Jaundice is more common in patients with effusive FIP [35]. Young cats can experience the inability to gain weight, stunting their growth [35].

A wide range of ocular lesions and neurological signs can be appreciated on physical examination. Ocular signs include conjunctivitis, mucopurulent ocular discharge, the thickening and hyperemia of the nictitans, uveitis with dyscoria or anisocoria, aqueous flare, keratic precipitates, hypopyon, hyphema, chorioretinitis, perivascular infiltrates, retinal detachment, and blindness [33]. Neurological signs may present themselves as behavioral changes, hyperreflexia, ataxia, nystagmus, or cranial nerve deficits [33].

### 2.3. Wet (Effusive) Form

The clinical manifestation of FIP is reflected by the distribution of vasculitis and (pyo)granulomatous lesions [5]. Effusive FIP (wet form) is characterized by fluid accumulation in the abdomen or chest, leading to difficulty while breathing, abdominal distension, and lethargy. Affected cats may also show fever, weight loss, and decreased appetite [13]. Vasculopathy can result in effusions (wet FIP). Rapid development and widespread vasculitis has seen the effusive form lead to fluid accumulation in the body cavity, particularly the abdomen [33]. Effusions in FIP cats commonly occur as ascites (often with abdominal distension) but can also manifest as pleural effusion (presenting with dyspnea), pericardial effusion (potentially presenting with heart failure), or scrotal effusion [5].

Interestingly, the American Association of Feline Practitioners (AAFP) states that distinguishing between the “wet” (effusive) and “dry” (non-effusive) forms of FIP may not be particularly helpful, as both represent different manifestations of the same underlying disease process [31]. The European Advisory Board on Cat Diseases (ABCD) has a similar stance, asserting that differentiating between “effusive” and “non-effusive” is important for diagnostic purposes, since effusion analysis is very useful [5]. Furthermore, the two forms of presentation frequently transition from one to the other, and they can even overlap so much as to prompt some scientists to consider a third form of the disease, a “mixed” form [34]. This “mixed” form of FIP is described as a transitional state from one of the effusive or non-effusive types to the other, in which symptoms of both types are observed at the same time [34]. Often, cats with non-effusive FIP will develop effusive signs later, and pyogranulomatous lesions are found at necropsy of cats who presented with effusive FIP [31].

## 3. Diagnosing FIP

Confirming FIP cases can be challenging, because clinical signs and symptoms are often non-specific and can mimic other diseases, so a tentative diagnosis must be approached with caution. Understanding each diagnostic test’s accuracy and relevance is crucial in diagnosing FIP, and the diagnosis must ultimately be tailored to each cat’s specific presentation [31]. At present, there is no single diagnostic test that can reliably diagnose every case of FIP [36]. A thorough physical exam can reveal signs such as abdominal distension, jaundice, and lymph node enlargement, and neurological exams may indicate brain or spinal involvement [32]. The literature suggests an algorithm for diagnosing FIP, which considers environmental factors, clinical signs, laboratory findings, serological tests, and viral antigen detection. Veterinarians may also use a combination of blood tests, imaging (such as radiographs and ultrasound), and an analysis of fluid accumulation to make a diagnosis. Various test modalities can be applied to different sample types, such as blood (whole blood, serum, plasma, or PBMCs), effusions (thoracic, abdominal, pericardial), tissues, cerebrospinal fluid (CSF), aqueous humor, and tissue fine-needle aspirates (FNAs) or biopsies [31]. In some cases, a biopsy may be required for confirmation [31]. Table 1 summarizes various diagnostic techniques for feline infectious peritonitis (FIP), including sample types, diagnostic method details, and pros/cons. This table includes tests that can be used individually or in combination, as FIP diagnosis typically requires a multifactorial approach due to the disease’s complex nature [31,35,37,38,39,40,41,42]. Recently, machine learning techniques were used to predict the rate of feline infectious peritonitis (FIP) diagnoses, with a specific focus on mutations in the spike protein gene of the feline coronavirus model obtained (CatBoost algorithm) [43].

## 4. Treatments and Management

The therapeutic approaches for feline infectious peritonitis (FIP) have advanced significantly in recent years, especially with the development of antiviral drugs. Main therapeutic options for FIP, include antiviral treatments, immunomodulatory approaches, and supportive care that are discussed below.

### 4.1. Antiviral Therapy—Nucleoside Analogs, Protease Inhibitors, and N-Protein Inhibitors

Antiviral drugs (Table 2) have revolutionized FIP treatment in recent years [44]. These drugs target the replication of the FIP virus (FIPV) in cats, significantly improving the survival rate, particularly when administered in the early stages of the disease. Antivirals include nucleoside analog and protease inhibitors

*Nucleoside analogs* like GS-441524, an antiviral related to remdesivir, interfere with the virus’s ability to replicate its RNA, thereby stopping viral proliferation [44]. This antiviral has shown high efficacy in treating FIP, especially in cases diagnosed early [44,45,46]. It is typically administered as an injectable drug but has also been formulated into oral capsules for ease of use. The treatment duration often lasts 12 weeks or more, depending on the severity of the disease [47]. Newer studies have demonstrated that treatment with oral GS-441524 for 42 days (six weeks) is sufficient to achieve complete remission in cats suffering from FIP [48]. Studies and clinical experience have also shown success rates as high as 80–90%, particularly for effusive (wet) FIP. Success rates are slightly lower in non-effusive (dry) FIP, which is more challenging to treat due to its granulomatous inflammation in organs [45]. One of the biggest challenges of GS-441524 is its long-term efficacy and safety are still under investigation [49]. However, both agents are currently not licensed and thus cannot be legally administered by veterinarians in many countries like Germany and Brazil. Legally, cats may only be legally treated with GS-441524 in a few countries (e.g., Great Britain and Australia) [50,51]. Another challenge relating to the lack of GS-441524 approval in many countries [52] is that it is often obtained through unofficial channels or prescribed off-label, resulting in high treatment costs [48,49,53].

Remdesivir (GS-5734), another antiviral originally developed for treating human RNA viruses like Ebola and later adapted for COVID-19 [54], is closely related to GS-441524 and works similarly by targeting viral RNA synthesis [55]. Some veterinarians have used Remdesivir as an alternative where GS-441524 is unavailable [51,56]. Though the research is less extensive in cats, Remdesivir has shown promising results, especially as an initial treatment followed by GS-441524 [55]. A 2023 study by Coggins et al. demonstrated that using remdesivir as an initial injectable treatment, followed by oral GS-441524, resulted in high survival rates (86%) in cats with FIP. Most cats showed significant improvement, with 56% achieving clinical remission by day 84. The cost of and access to Remdesivir may vary depending on the region, and it is sometimes reserved for cats that do not respond well to GS-441524 [57].

Molnupiravir (EIDD-2801), developed by Merck, is another nucleoside analog currently authorized by the FDA under an Emergency Use Authorization (EUA) for treating COVID-19 in adults [58,59]. This oral prodrug of the nucleoside analog β-D-N4-hydroxycytidine induces mutations by converting guanine to adenine and cytosine to uracil in viral RNA [58,60]. This mechanism elevates the mutation rate past a tolerable threshold, ultimately deactivating the virus. Though still in experimental stages for FIP, Molnupiravir has shown promise [58,61,62], especially in cases resistant to GS-441524 [52]. Some veterinarians are beginning to explore its use as a complementary or alternative therapy, but formal studies in cats are still limited [34,63].

*Protease Inhibitors (GC376 and GC373)*—The coronavirus main protease (M^pro^, also called 3CL^pro^) has an essential role in mediating viral replication and transcription and is therefore an attractive target for drug development [64]. GC373 (Table 2) and prodrug GC376 (a dipeptidyl aldehyde bisulfite adduct) are protease inhibitors that have shown promise in inhibiting the replication of the virus in laboratory settings [65,66]. GC373 and GC376 share structural similarities, as indicated by their chemical frameworks [64]. Clinical trials are needed to assess their effectiveness and safety in real-world cases [46,67].

*N protein* inhibitors—The FIP virion comprises three distinct protein species: the spike protein (S) with a molecular weight of 200,000, the membrane glycoprotein (M) weighing 25,000–30,000, and the nucleocapsid protein (N) with a molecular weight of 45,000 [68]. Researchers explored FIPV immunization using M and N proteins, showing 84.7% and 77% identity with porcine gastroenteritis virus. Vaccinia virus recombinants elicited antibodies in kittens. Antibody-dependent enhancement appeared to be S protein-specific. The N protein had no effect, but M protein immunization improved survival rates (3/8 vs. 1/8 in controls) [69]. Further recent findings revealed that K31 (Table 2), a broad-spectrum coronavirus N protein inhibitor, effectively suppressed the replication of feline infectious peritonitis virus (FIPV) in cell culture [70]. K31 interacts with the N protein of FIPV, preventing its binding to viral genomic RNA. A single dose of K31 reduced FCoV replication to undetectable levels within 24 h of treatment. While K31 does not interfere with viral entry into host cells, it inhibits post-entry steps of viral replication. Further research is required to evaluate its antiviral efficacy in FIPV-infected cats.

**Table 2:** Therapeutic and Management Approaches for feline infectious peritonitis (FIP).

Category	Treatment/Approach	Mechanism	Limitations and Status
**Nucleoside Analogs**	GS-441524	Nucleoside analog that inhibits viral RNA replication; high efficacy especially in early-stage wet FIP [44,45,46,47,48]	Not licensed in many countries [49,50,51]; often sourced unofficially; costly [52,53]
	Remdesivir (GS-5734)	Related to GS-441524; inhibits viral RNA synthesis; used where GS-441524 is unavailable [51,54,55,56]	Limited feline data; cost and regional access issues [57]
	Molnupiravir (EIDD-2801)	Increases mutation rate of viral RNA, leading to viral inactivation [58,59,60]; shows promise in GS-resistant cases [61,62,63]	Experimental use in cats; more studies required [34,58,63]
**Protease Inhibitors**	GC376/GC373	Inhibit coronavirus main protease (3CLpro); block replication [64,65,66]	Clinical trial data needed to confirm efficacy and safety [46,47,48,49,50,51,52,53,54,55,56,57,58,59,60,61,62,63,64,65,66,67]
**N Protein Inhibitors**	K31	Binds to viral N protein; blocks RNA binding; halts replication post-entry [70]	Demonstrated in vitro only; in vivo feline data pending
**Monoclonal Antibodies**	FIPV-infected cell antibodies (FICA), Anti-TNF-α mAb	Neutralize infected cells and reduce inflammatory cytokine effects [71,72]	Research ongoing; safety and efficacy under evaluation [73]
**Immunomodulatory Drugs**	Polyprenyl Immunostimulant (PI)	Boosts innate immune function; mixed results in dry FIP [74,75,76]	Variable outcomes; still experimental
	Glucocorticoids, Propentofylline	Used to manage inflammation and symptoms [77]	May suppress immunity; controversial in viral infections [77,78]
**Supportive Care**	General Management	Includes hydration, nutrition, and secondary infection control	Symptomatic only; does not affect viral replication
**Vaccination**	Pfizer intranasal vaccine	S protein-based; controversial efficacy; risk of antibody-dependent enhancement [79,80,81,82]	Not widely recommended due to potential disease exacerbation
	mRNA-based vaccine (N protein)	CG-optimized, lipid nanoparticle-encapsulated mRNA vaccine; shows in vitro stability and murine immune activation [83]	Preclinical stage; feline in vivo safety trials pending

### 4.2. Monoclonal Antibodies (FIPV-Infected Cell Antibodies—FICA)

Monoclonal antibodies that specifically target FIP-infected cells have been explored [71]. These antibodies aim to neutralize the virus and stimulate an immune response [71]. In some studies, anti-TNF-α antibody (Table 2) has been used to block TNF-α that is involved in exacerbating clinical signs of FIP [72], and research is ongoing to determine its efficacy and safety [73].

### 4.3. Immunomodulatory Drugs

Drugs that help modulate the immune response were considered in FIP management. Examples include polyprenyl immunostimulant (PI) [74] (Table 2), which was used to boost the cat’s immune system experimentally, and some veterinarians have reported positive outcomes [75]. However, its effectiveness is variable, and more research is needed [75]. In another study two out of three cats with the dry form of FIP treated with PI were alive two years after diagnosis but still on treatment and a third survived for 14 months with a 4.5-month treatment [76].

Symptomatic anti-inflammatory medications like glucocorticoids and propentofylline may be used to manage the inflammatory response associated with FIP [77]. These drugs help reduce inflammation but do not directly target the virus [78]. However, their use is controversial, as they may suppress the immune system, and the benefits must be carefully weighed against potential risks [77].

### 4.4. Vaccines

**Figure 1:**
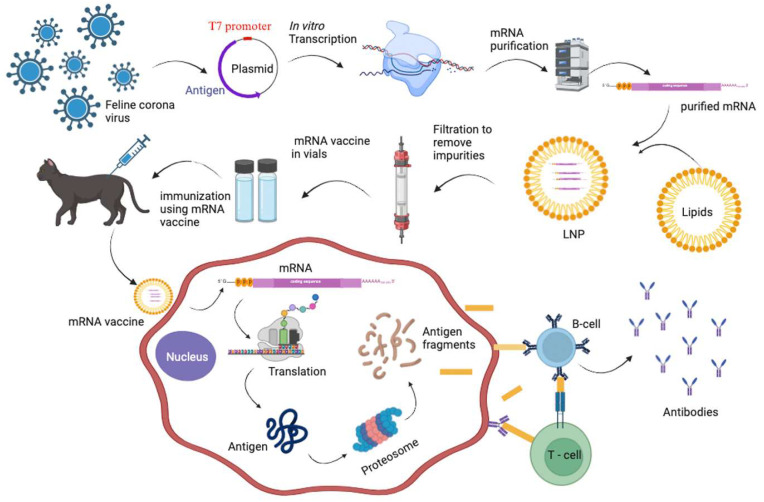
mRNA vaccine development for feline coronavirus.

While there is currently no recommended available vaccine for FIP, researchers have been working on developing vaccines that can provide immunity against feline coronavirus [79,80,83]. There is a single commercial vaccine from Pfizer which is an intranasal vaccine; however, its risks and benefits present a complicated issue [81]. Various vaccines aimed at preventing FIPV infection have been found to worsen the disease, likely due to immune enhancement caused by virus-specific immunoglobulins targeting the outer envelope (S) protein [82]. Studies suggest that the protective immune response involves a mechanism other than humoral immunity consisting of FIPV-neutralizing antibodies [82]. Since the COVID-19 pandemic, vaccine development strategies have shifted to mRNA platforms. Recently, a lipid nanoparticle-encapsulated mRNA encoding the nucleocapsid (N) of FCoV (Table 2) were developed and found to be stable in vitro and capable of eliciting an immune response [83]. This study presents the initial development of a CG-optimized LNP-encapsulated mRNA vaccine for FIP, showing sustained protein production in feline cells for at least a week in vitro. In mice, the vaccine elicited specific humoral and cellular responses after a prime-boost strategy, with no safety concerns despite variability in immune responses. These findings support advancing to in vivo safety and immunogenicity studies in felines. The development of mRNA vaccines is a rapid process that begins with identifying a target antigen (Figure 1).

Vaccinia virus recombinants elicited antibodies in kittens. For FCoV, the S-protein is often selected despite its association with antibody-dependent enhancement (ADE), where non-neutralizing antibodies can trigger infection. Alternatively, the N-protein of FCoV is another promising antigen target. Although one study elucidated that the N protein had no effect, M protein immunization improved survival rates (3/8 vs. 1/8 in controls) [69]. Once the antigen is chosen, the DNA encoding it is cloned into a plasmid downstream of a T7 promoter. mRNA synthesis is then performed using an in vitro T7 transcription reaction (Figure 1). The synthetic mRNA is chemically modified and purified to eliminate misfolded mRNA molecules. It is then encapsulated in lipid nanoparticles (LNPs), with further filtration to remove unincorporated mRNA or empty LNPs. The purified LNPs are used to immunize the host. Inside the host cells, the mRNA is translated into the antigen by the cell’s translation machinery. The resulting antigen is processed by the proteasome and presented to the immune system by antigen-presenting cells. FCoV N-protein-derived antigens produced through mRNA vaccination have been shown to elicit both B-cell and T-cell immune responses. Following this, preclinical testing evaluates the vaccine, and subsequent clinical trials assess safety (Phase 1), determine optimal dosing and immune response (Phase 2), and confirm safety and efficacy in larger populations (Phase 3) [83].

This diagram illustrates the creation and immune response of an mRNA vaccine. The process includes synthesizing mRNA from viral antigen genes, encapsulating it in lipid nanoparticles (LNPs), and administering it as a vaccine. Once inside cells, the mRNA is translated into an antigen, triggering the immune system to produce antibodies and activate T-cells, providing protection against future infections.

## 5. Prevention

Preventing feline infectious peritonitis (FIP) involves reducing exposure to feline coronavirus (FCoV), the virus responsible for the disease [36]. While the complete elimination of risk is not always feasible, several effective strategies can significantly lower the likelihood of transmission. Maintaining a clean environment is essential. Regularly disinfect litter boxes, food and water bowls, and living areas using agents known to kill coronaviruses. Good hygiene practices—such as thoroughly washing hands after handling multiple cats, particularly those from different households—are also crucial [84,85]. Limiting cat population density plays an important role. Avoid overcrowded living conditions, as these increase the chances of FCoV spread. Providing adequate space for each cat reduces stress and minimizes territorial conflict, both of which can contribute to viral transmission [86]. When introducing new cats into a multi-cat household, it is advisable to test them for FCoV and isolate them temporarily to monitor for signs of illness. Keeping infected cats separate from healthy ones helps contain the virus and protect the broader feline community [5]. Reducing environmental and social stress is another key preventive measure. Stress is a known factor in the progression of FCoV to FIP. Ensuring a stable, low-stress environment—with consistent routines and a comfortable living space—can support immune health and reduce disease risk [87]. Regular veterinary check-ups are vital for early detection of health issues. Any changes in appetite, behavior, or general condition should be promptly addressed [88]. Responsible breeding practices are also critical, and breeders should avoid mating cats known to carry the mutated form of FCoV to help limit its spread [31,84,89].

## 6. Conclusions

The journey from the challenge to cure of feline infectious peritonitis highlights remarkable advancements in veterinary science. Once considered uniformly fatal, FIP has transitioned into a manageable disease, thanks to novel antiviral therapies such as GS-441524 and remdesivir. These treatments have demonstrated high efficacy, leading to remission in most cases when administered under proper protocols. Despite these successes, challenges remain. Vaccines for FIP have had limited effectiveness due to the complexity of FCoV mutation and immune response. Research continues to refine vaccine strategies, aiming to prevent FCoV infections and reduce the likelihood of FIP development. Recent advancements in mRNA vaccines, which have shown promise in other infectious diseases like COVID-19, are now being explored for their potential to generate targeted immune responses against FCoV, the precursor of FIP. These vaccines work by delivering genetic instructions that enable the cat’s cells to produce viral proteins, prompting an immune response without exposure to the live virus. Continued research will determine the viability and widespread applicability of these vaccines as part of comprehensive FIP control strategies.

Preventative measures like responsible breeding, hygiene in multi-cat households, and stress reduction also play critical roles in controlling the disease. As treatments and vaccines evolve, the veterinary community moves closer to making FIP not only treatable but preventable, offering hope to cats and their caregivers worldwide.

## Figures and Tables

**Table 1:** Different diagnostic techniques for feline infectious peritonitis (FIP).

Diagnostic Technique	Sample Type	Method	Pros	Cons
**Clinical Signs and History**	Clinical exam	Assessment of symptoms and risk factors	Quick initial indication of FIP presence	Non-specific; symptoms overlap with other diseases
**Serology (Antibody Testing)**	Blood (serum, plasma)	Detects antibodies to feline coronavirus (FCoV)	Useful for screening in high-risk groups	High incidence of false positives; does not distinguish FIP from FCoV
**RT-PCR (Reverse Transcription PCR)**	Blood, effusion, CSF	Detects viral RNA; can target FCoV mutations	High sensitivity; mutation analysis for FIP strains	Expensive; false negatives if viral load is low
**Immunohistochemistry (IHC)**	Tissue biopsies, FNA	Identifies viral antigen in tissue cells	Confirmatory for FIP diagnosis	Requires tissue biopsy; invasive; expertise needed
**Immunocytochemistry (ICC)**	Effusions, FNAs	Stains cells to detect viral Proteins in effusions	Specific for FIP-associated proteins	Limited to effusive cases; Expertise required
**Rivalta Test**	Effusions (abdomen, thorax)	Tests effusions for exudate characteristics	Quick and inexpensive; often used in clinics	Less accurate; false negatives possible
**Histopathology**	Tissue biopsy	Examines tissue for characteristic FIP lesions	Highly specific with pathologist expertise	Invasive; requires anesthesia; time-consuming
**Hemogram and Biochemistry**	Blood	Measures white blood cells, globulin, etc.	Commonly available; may support FIP suspicion	Non-specific; may overlap with other inflammatory diseases
**Albumin-to-Globulin Ratio**	Blood	Measures protein levels in blood	Simple and cost-effective screening method	Not confirmatory; low specificity
**CSF Analysis**	Cerebrospinal fluid	Analyzes CSF for cell count and protein levels	Useful for neurological FIP cases	Invasive; requires anesthesia; only useful in neurological FIP

## Data Availability

No new data were created or analyzed in this study.

## References

[1] Holzworth J. (1963). Some important disorders of cats. Cornell Vet..

[2] Masters P.S. (2006). The molecular biology of coronaviruses. Adv. Virus Res..

[3] Kennedy M., Citino S., McNabb A.H., Moffatt A.S., Gertz K., Kania S. (2002). Detection of Feline Coronavirus in Captive Felidae in the USA. J. Vet. Diagn. Investig..

[4] Pedersen N.C., Evermann J.F., McKeirnan A.J., Ott R.L. (1984). Pathogenicity studies of feline coronavirus isolates 79-1146 and 79-1683. Am. J. Vet. Res..

[5] Tasker S., Addie D.D., Egberink H., Hofmann-Lehmann R., Hosie M.J., Truyen U., Belák S., Boucraut-Baralon C., Frymus T., Lloret A. (2023). Feline Infectious Peritonitis: European Advisory Board on Cat Diseases Guidelines. Viruses.

[6] Sherding R.G. (2006). Feline Infectious Peritonitis (Feline Coronavirus).

[7] Vennema H., Poland A., Foley J., Pedersen N.C. (1998). Feline infectious peritonitis viruses arise by mutation from endemic feline enteric coronaviruses. Virology.

[8] Stranieri A., Probo M., Pisu M.C., Fioletti A., Meazzi S., E Gelain M., Bonsembiante F., Lauzi S., Paltrinieri S. (2019). Preliminary investigation on feline coronavirus presence in the reproductive tract of the tom cat as a potential route of viral transmission. J. Feline Med. Surg..

[9] Pedersen N.C., Black J.W., Boyle J.F., Evermann J.F., McKeirnan A.J., Ott R.L. (1984). Pathogenic differences between various feline coronavirus isolates. Adv. Exp. Med. Biol..

[10] Dye C., Siddell S.G. (2005). Genomic RNA sequence of Feline coronavirus strain FIPV WSU-79/1146. J. Gen. Virol..

[11] Tresnan D.B., Levis R., Holmes K.V. (1996). Feline aminopeptidase N serves as a receptor for feline, canine, porcine, and human coronaviruses in serogroup I. J. Virol..

[12] Gao Y.-Y., Wang Q., Liang X.-Y., Zhang S., Bao D., Zhao H., Li S.-B., Wang K., Hu G.-X., Gao F.-S. (2023). An updated review of feline coronavirus: Mind the two biotypes. Virus Res..

[13] Pedersen N.C. (2009). A review of feline infectious peritonitis virus infection: 1963–2008. J. Feline Med. Surg..

[14] Gross J.L., Behrens D.L., Mullins D.E., Kornblith P.L., Dexter D.L. (1988). Plasminogen activator and inhibitor activity in human glioma cells and modulation by sodium butyrate. Cancer Res..

[15] Kipar A., Bellmann S., Kremendahl J., Köhler K., Reinacher M. (1998). Cellular composition, coronavirus antigen expression and production of specific antibodies in lesions in feline infectious peritonitis. Vet. Immunol. Immunopathol..

[16] Kipar A., Baptiste K., Barth A., Reinacher M. (2006). Natural FCoV infection: Cats with FIP exhibit significantly higher viral loads than healthy infected cats. J. Feline Med. Surg..

[17] Pedersen N.C. (2014). An update on feline infectious peritonitis: Virology and immunopathogenesis. Vet. J..

[18] Cornelissen E., Dewerchin H.L., Van Hamme E., Nauwynck H.J. (2009). Absence of antibody-dependent, complement-mediated lysis of feline infectious peritonitis virus-infected cells. Virus Res..

[19] Dewerchin H.L., Desmarets L.M., Noppe Y., Nauwynck H.J. (2014). Myosins 1 and 6, myosin light chain kinase, actin and microtubules cooperate during antibody-mediated internalisation and trafficking of membrane-expressed viral antigens in feline infectious peritonitis virus infected monocytes. Vet. Res..

[20] Kipar A., Meli M.L., Failing K., Euler T., Gomes-Keller M.A., Schwartz D., Lutz H., Reinacher M. (2006). Natural feline coronavirus infection: Differences in cytokine patterns in association with the outcome of infection. Vet. Immunol. Immunopathol..

[21] Tekes G., Spies D., Bank-Wolf B., Thiel V., Thiel H.-J. (2012). A Reverse Genetics Approach To Study Feline Infectious Peritonitis. J. Virol..

[22] Regan A.D., Ousterout D.G., Whittaker G.R. (2010). Feline Lectin Activity Is Critical for the Cellular Entry of Feline Infectious Peritonitis Virus. J. Virol..

[23] Poland A.M., Vennema H., E Foley J., Pedersen N.C. (1996). Two related strains of feline infectious peritonitis virus isolated from immunocompromised cats infected with a feline enteric coronavirus. J. Clin. Microbiol..

[24] Tekes G., Thiel H.-J. (2016). Pathognesis of feline infectious peritonitis. Adv. Virus Res..

[25] Addie D.D., Toth S., Murray G.D., Jarrett O. (1995). Risk of feline infectious peritonitis in cats naturally infected with feline coronavirus. Am. J. Vet.-Res..

[26] Rottier P.J.M., Nakamura K., Schellen P., Volders H., Haijema B.J. (2005). Acquisition of Macrophage Tropism during the Pathogenesis of Feline Infectious Peritonitis Is Determined by Mutations in the Feline Coronavirus Spike Protein. J. Virol..

[27] Ouyang H., Liu J., Yin Y., Cao S., Yan R., Ren Y., Zhou D., Li Q., Li J., Liao X. (2022). Epidemiology and Comparative Analyses of the S Gene on Feline Coronavirus in Central China. Pathogens.

[28] Brown M.A. (2011). Genetic determinants of pathogenesis by feline infectious peritonitis virus. Vet. Immunol. Immunopathol..

[29] Stoddart A.C., Scott F.W. (1989). Intrinsic resistance of feline peritoneal macrophages to coronavirus infection correlates with in vivo virulence. J. Virol..

[30] Sykes J.E. (2014). Canine and Feline Infectious Diseases.

[31] Thayer V., Gogolski S., Felten S., Hartmann K., Kennedy M., Olah G.A. (2022). 2022 AAFP/EveryCat Feline Infectious Peritonitis Diagnosis Guidelines. J. Feline Med. Surg..

[32] Kennedy M.A. (2020). Feline Infectious Peritonitis: Update on Pathogenesis, Diagnostics and Treatment. Veterinary Clinics of North America: Small Animal Practice.

[33] Doli T., Ohno M., Kaku M., Odani S., To K., Takano T. (2025). Development of rapid and simple FCoV RNA detection systems using Rt-PCR and RT-RPA combined with STH-PAS to diagnose FP in cats. J. Virol. Methgods.

[34] Katayama M., Uemura Y., Katori D. (2024). Effect of Nucleic Acid Analog Administration on Fluctuations in the Albumin-to-Globulin Ratio in Cats with Feline Infectious Peritonitis. Animals.

[35] Tasker S. (2018). Diagnosis of feline infectious peritonitis: Update on evidence supporting available tests. J. Feline Med. Surg..

[36] Addie D., Belák S., Boucraut-Baralon C., Egberink H., Frymus T., Gruffydd-Jones T., Hartmann K., Hosie M.J., Lloret A., Lutz H. (2009). Feline Infectious Peritonitis: ABCD Guidelines on Prevention and Management. J. Feline Med. Surg..

[37] Felten S., Hartmann K. (2019). Diagnosis of Feline Infectious Peritonitis: A Review of the Current Literature. Viruses.

[38] Hartmann K. (2005). Feline infectious peritonitis. Vet. Clin. N. Am. Small. Anim. Pract..

[39] Kipar A., Meli M.L. (2014). Feline infectious peritonitis: Still an enigma?. Vet. Pathol..

[40] Fischer Y., Sauter-Louis C., Hartmann K. (2012). Diagnostic accuracy of theRivalta test for feline infectious peritonitis. Vet. Clin. Pathol..

[41] Pedersen N.C. (2014). An update on feline infectious peritonitis: Diagnostics and therapeutics. Vet. J..

[42] Addie D.D., le Poder S., Burr P., Decaro N., Graham E., Hofmann-Lehmann R., Jarrett O., McDonald M., Meli M.L. (2014). Utility of feline coronavirus antibody tests. J. Feline Med. Surg..

[43] Kuo P.-H., Li Y.-H., Yau H.-T. (2024). Development of feline infectious peritonitis diagnosis system by using CatBoost algorithm. Comput. Biol. Chem..

[44] Pedersen N.C., Perron M., Bannasch M., Montgomery E., Murakami E., Liepnieks M., Liu H. (2019). Efficacy and safety of the nucleoside analog GS-441524 for treatment of cats with naturally occurring feline infectious peritonitis. J. Feline Med. Surg..

[45] Dickinson P.J., Bannasch M., Thomasy S.M., Murthy V.D., Vernau K.M., Liepnieks M., Montgomery E., Knickelbein K.E., Murphy B., Pedersen N.C. (2020). Antiviral treatment using the adenosine nucleoside analogue GS-441524 in cats with clinically diagnosed neurological feline infectious peritonitis. J. Vet. Intern. Med..

[46] Mulligan A.J., Browning M.E. (2024). Quality assessment and characterization of unregulated antiviral drugs for feline infectious peritonitis: Implications for treatment, safety, and efficacy. Am. J. Vet.-Res..

[47] Zwicklbauer K., Krentz D., Bergmann M., Felten S., Dorsch R., Fischer A., Hofmann-Lehmann R., Meli M.L., Spiri A.M., Alberer M. (2023). Long-term follow-up of cats in complete remission after treatment of feline infectious peritonitis with oral GS-441524. J. Feline Med. Surg..

[48] Zuzzi-Krebitz A.-M., Buchta K., Bergmann M., Krentz D., Zwicklbauer K., Dorsch R., Wess G., Fischer A., Matiasek K., Hönl A. (2024). Short Treatment of 42 Days with Oral GS-441524 Results in Equal Efficacy as the Recommended 84-Day Treatment in Cats Suffering from Feline Infectious Peritonitis with Effusion—A Prospective Randomized Controlled Study. Viruses.

[49] Jones S., Novicoff W., Nadeau J., Evans S. (2021). Unlicensed GS-441524-Like Antiviral Therapy Can Be Effective for at-Home Treatment of Feline Infectious Peritonitis. Animals.

[50] Krentz D., Bergmann M., Felten S., Hartmann K. (2023). Options for treatment of feline infectious peritonitis- previously and today. Tierarztl Prax. Ausg. K Kleintiere Heimtiere.

[51] Taylor S.S., Coggins S., Barker E.N., Gunn-Moore D., Jeevaratnam K., Norris J.M., Hughes D., Stacey E., MacFarlane L., O’bRien C. (2023). Retrospective study and outcome of 307 cats with feline infectious peritonitis treated with legally sourced veterinary compounded preparations of remdesivir and GS-441524 (2020–2022). J. Feline Med. Surg..

[52] Sase O., Iwami T., Sasaki T., Sano T. (2024). GS-441524 and molnupiravir are similarly effective for the treatment of cats with feline infectious peritonitis. Front. Vet. Sci..

[53] Kent A.M., Guan S., Jacque N., Novicoff W., Evans S.J.M. (2024). Unlicensed antiviral products used for the at-home treatment of feline infectious peritonitis contain GS-441524 at significantly different amounts than advertised. J. Am. Vet. Med. Assoc..

[54] Radoshitzky S.R., Iversen P., Lu X., Zou J., Kaptein S.J.F., Stuthman K.S., Van Tongeren S.A., Steffens J., Gong R., Truong H. (2023). Expanded profiling of Remdesivir as a broad- spectrum antiviral and low potential for interaction with other medications in vitro. Sci. Rep..

[55] Coggins S.J., Norris J.M., Malik R., Govendir M., Hall E.J., Kimble B., Thompson M.F. (2023). Outcomes of treatment of cats with feline infectious peritonitis using parenterally administered remdesivir, with or without transition to orally administered GS-441524. J. Vet. Intern. Med..

[56] Green J., Syme H., Tayler S. (2023). Thirty-two cats with effusive or non-effusive feline infectious peritonitis treated with a combination of remdesivir and GS-441524. J. Vet. Intern. Med..

[57] Cosaro E., Pires J., Castillo D., Murphy B.G., Reagan K.L. (2023). Efficacy of Oral Remdesivir Compared to GS-441524 for Treatment of Cats with Naturally Occurring Effusive Feline Infectious Peritonitis: A Blinded, Non-Inferiority Study. Viruses.

[58] Roy M., Jacque N., Novicoff W., Li E., Negash R., Evans S.J.M. (2022). Unlicensed Molnupiravir is an Effective Rescue Treatment Following Failure of Unlicensed GS-441524-like Therapy for Cats with Suspected Feline Infectious Peritonitis. Pathogens.

[59] Khoo S.H., Fitzgerald R., Fletcher T., Ewings S., Jaki T., Lyon R., Downs N., Walker L., Tansley-Hancock O., Greenhalf W. (2021). Optimal dose and safety of molnupiravir in patients with early SARS-CoV-2: A Phase I, open-label, dose-escalating, randomized controlled study. J. Antimicrob. Chemother..

[60] Singh A.K., Singh A., Singh R., Misra A. (2021). Molnupiravir in COVID-19: A systematic review of literature. Diabetes Metab. Syndr..

[61] Sase O. (2023). Molnupiravir treatment of 18 cats with feline infectious peritonitis: A case series. J. Vet. Intern. Med..

[62] Barua S., Kaltenboeck B., Juan Y.-C., Bird R.C., Wang C. (2023). Comparative Evaluation of GS-441524, Teriflunomide, Ruxolitinib, Molnupiravir, Ritonavir, and Nirmatrelvir for In Vitro Antiviral Activity against Feline Infectious Peritonitis Virus. Vet. Sci..

[63] Reagan K.L., Brostoff T., Pires J., Rose A., Castillo D., Murphy B.G. (2024). Open label clinical trial of orally administered molnupiravir as a first-line treatment for naturally occurring effusive feline infectious peritonitis. J. Vet. Intern. Med..

[64] Lu J., Chen S.A., Khan M.B., Brassard R., Arutyunova E., Lamer T., Vuong W., Fischer C., Young H.S., Vederas J.C. (2022). Crystallization of Feline Coronavirus M(pro) With GC376 Reveals Mechanism of Inhibition. Front. Chem..

[65] Kim Y., Lovell S., Tiew K.C., Mandadapu S.R., Alliston K.R., Battaile K.P., Groutas W.C., Chang K.O. (2012). Broad-spectrum antivirals against 3C or 3C-like proteases of picornaviruses, noroviruses, and coronaviruses. J. Virol..

[66] Kim Y., Shivanna V., Narayanan S., Prior A.M., Weerasekara S., Hua D.H., Kankanamalage A.C.G., Groutas W.C., Chang K.-O., Perlman S. (2015). Broad-Spectrum Inhibitors against 3C-Like Proteases of Feline Coronaviruses and Feline Caliciviruses. J. Virol..

[67] Perera K.D., Rathnayake A.D., Liu H., Pedersen N.C., Groutas W.C., Chang K.-O., Kim Y. (2019). Characterization of amino acid substitutions in feline coronavirus 3C-like protease from a cat with feline infectious peritonitis treated with a protease inhibitor. Vet. Microbiol..

[68] Spaan W., Cavanagh D., Horzinek M.C. (1988). Coronaviruses: Structure and Genome Expression. J. Gen. Virol..

[69] Vennema H., De Groot R.J., Harbour D.A., Horzinek M.C., Spaan W.J. (1991). Primary structure of the membrane and nucleocapsid protein genes of feline infectious peritonitis virus and immunogenicity of recombinant vaccinia viruses in kittens. Virology.

[70] Mohseni N., Royster A., Ren S., Ma Y., Pintado M., Mir M., Mir S. (2023). A novel compound targets the feline infectious peritonitis virus nucleocapsid protein and inhibits viral replication in cell culture. J. Biol. Chem..

[71] Doki T., Takano T., Kawagoe K., Kito A., Hohdatsu T. (2016). Therapeutic effect of anti-feline TNF-alpha monoclonal antibody for feline infectious peritonitis. Res. Vet. Sci..

[72] Takano T., Azuma N., Satoh M., Toda A., Hashida Y., Satoh R., Hohdatsu T. (2009). Neutrophil survival factors (TNF-alpha, GM-CSF, and G-CSF) pro-duced by macrophages in cats infected with feline infectious peritonitis virus contribute to the pathogenesis of granulomatous lesions. Arch. Virol..

[73] Doki T., Toda M., Hasegawa N., Hohdatsu T., Takano T. (2020). Therapeutic effect of an anti-human-TNF-alpha antibody and itraconazole on feline infectious peritonitis. Arch. Virol..

[74] Legendre A.M., Kuritz T., Galyon G., Baylor V.M., Heidel R.E. (2017). Polyprenyl Immunostimulant Treatment of Cats with Presumptive Non-Effusive Feline Infectious Peritonitis In a Field Study. Front. Vet. Sci..

[75] Černá P., Ayoob A., Baylor C., Champagne E., Hazanow S., Heidel R.E., Wirth K., Legendre A.M., Gunn-Moore D.A. (2022). Retrospective Survival Analysis of Cats with Feline Infectious Peritonitis Treated with Polyprenyl Immunostimulant That Survived over 365 Days. Pathogens.

[76] Legendre A.M., Bartges J.W. (2009). Effect of Polyprenyl Immunostimulant on the survival times of three cats with the dry form of feline infectious peritonitis. J. Feline Med. Surg..

[77] Hartmann K., Ritz S. (2008). Treatment of cats with feline infectious peritonitis. Vet. Immunol. Immunopathol..

[78] Kameshima S., Kimura Y., Doki T., Takano T., Park C.-H., Itoh N. (2020). Clinical efficacy of combination therapy of itraconazole and prednisolone for treating effusive feline infectious peritonitis. J. Vet. Med Sci..

[79] Hohdatsu T., Yamato H., Ohkawa T., Kaneko M., Motokawa K., Kusuhara H., Kaneshima T., Arai S., Koyama H. (2003). Vaccine efficacy of a cell lysate with recombinant baculovirus-expressed feline infectious peritonitis (FIP) virus nucleocapsid protein against progression of FIP. Vet. Microbiol..

[80] Fehr D., Holznagel E., Bolla S., Hauser B., Herrewegh A.A., Horzinek M.C., Lutz H. (1997). Placebo-controlled evaluation of a modified life virus vaccine against feline infectious peritonitis: Safety and efficacy under field conditions. Vaccine.

[81] Gerber J., Ingersoll J., Gast A., Christianson K., Selzer N., Landon R., Pfeiffer N., Sharpee R., Beckenhauer W. (1990). Protection against feline infectious peritonitis by intranasal inoculation of a temperature-sensitive FIPV vaccine. Vaccine.

[82] Wasmoen T.L., Kadakia N.P., Unfer R.C., Fickbohm B.L., Cook C.P., Chu H.-J., Acree W.M. (1995). Protection of cats from infectious peritonitis by vaccination with a recombinant raccoon poxvirus expressing the nucleocapsid gene of feline infectious peritonitis virus. Adv. Exp. Med. Biol..

[83] Brostoff T., Savage H.P., Jackson K.A., Dutra J.C., Fontaine J.H., Hartigan-O’connor D.J., Carney R.P., Pesavento P.A. (2024). Feline Infectious Peritonitis mRNA Vaccine Elicits Both Humoral and Cellular Immune Responses in Mice. Vaccines.

[84] Addie D.D. (2019). Feline infectious peritonitis: Answers to frequently asked questions concerning FIP and coronavirus. Vet. Nurs. J..

[85] Xiao S., Yuan Z., Huang Y. (2022). Disinfectants against SARS-CoV-2: A Review. Viruses.

[86] Sherding R.G. (2009). Feline Infectious Peritonitis (Feline Coronavirus). Saunders Man. Small Anim. Pract. May.

[87] Griffin B., Little S.E. (2012). Population Wellness: Keeping Cats Physically and Behaviorally Healthy. The Cat: Clinical Medicine and Management.

[88] Taylor S., Chan D.L., Villaverde C., Ryan L., Peron F., Quimby J., O’bRien C., Chalhoub S. (2022). 2022 ISFM Consensus Guidelines on Management of the Inappetent Hospitalised Cat. J. Feline Med. Surg..

[89] Addie D.D., Paltrinieri S., Pedersen N.C. (2004). Secong international feline coronavirus/feline infectious peritonitis s. Recommendations from work-shops of the second international feline coronavirus/feline infectious peritonitis symposium. J. Feline. Med. Surg..

